# Supplementary material for: DMRforPairs: identifying Differentially Methylated Regions between unique samples using array based methylation profiles
Source: BMC Bioinformatics. 2014 May 15;15:141. doi: 10.1186/1471-2105-15-141 (PMC4046028; doi:10.1186/1471-2105-15-141)
Supplement: Additional file 2 — DMRforPairs output for the comparison of A431-MCF7 and NA17018-NA17105. Please start from the HTML files in each folder. Available via the BMC Bioinformatics website. [file 1471-2105-15-141-S2.zip › 1394847754114233_MOESM2_ESM/A431_MCF7/figures/16033.pdf]

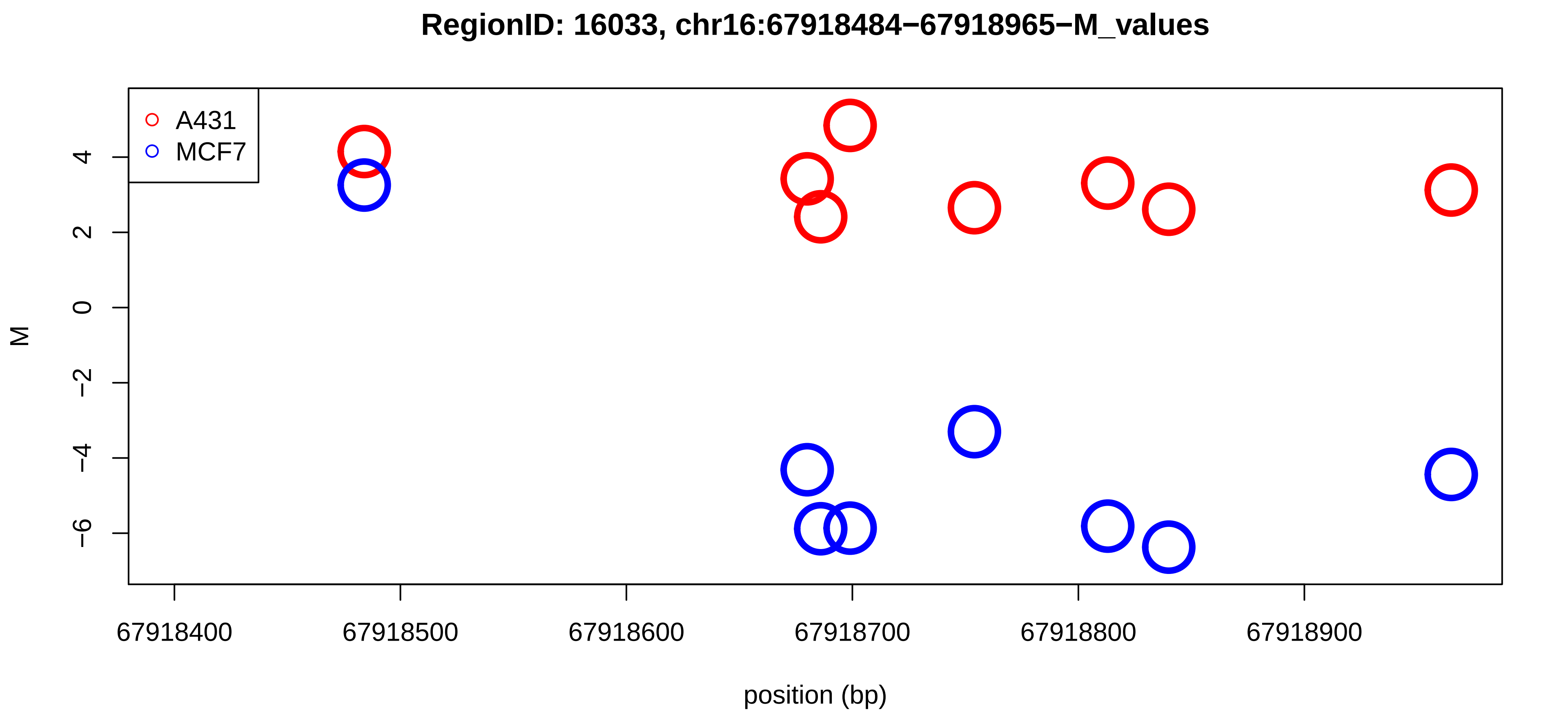

RegionID: 16033, chr16:67918484−67918965−M_values
A431
MCF7
M
4
2
0
−2
−4
−6
67918400
67918500
67918600
67918700
67918800
67918900
position (bp)

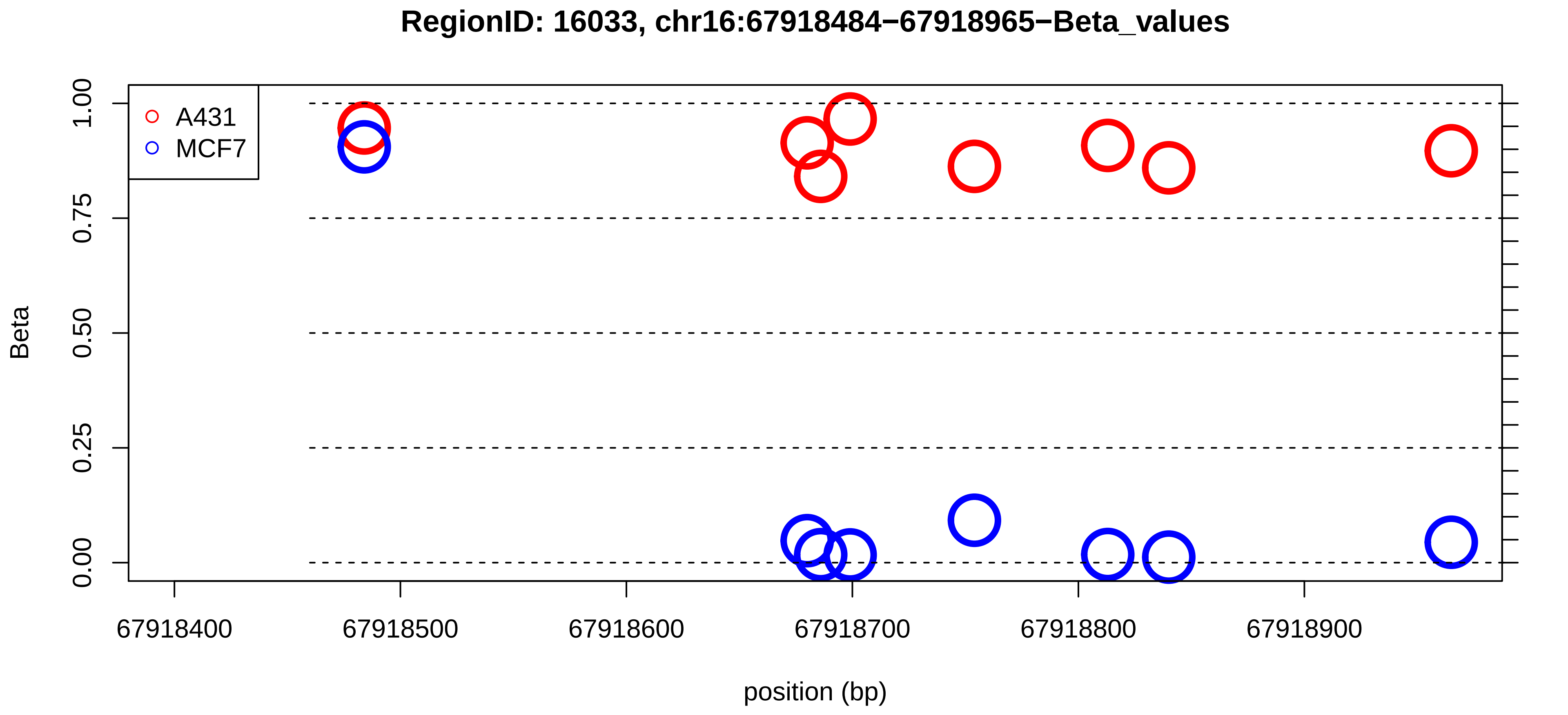

RegionID: 16033, chr16:67918484−67918965−Beta_values
A431
MCF7
Beta
1.00
0.75
0.50
0.25
0.00
67918400
67918500
67918600
67918700
67918800
67918900
position (bp)

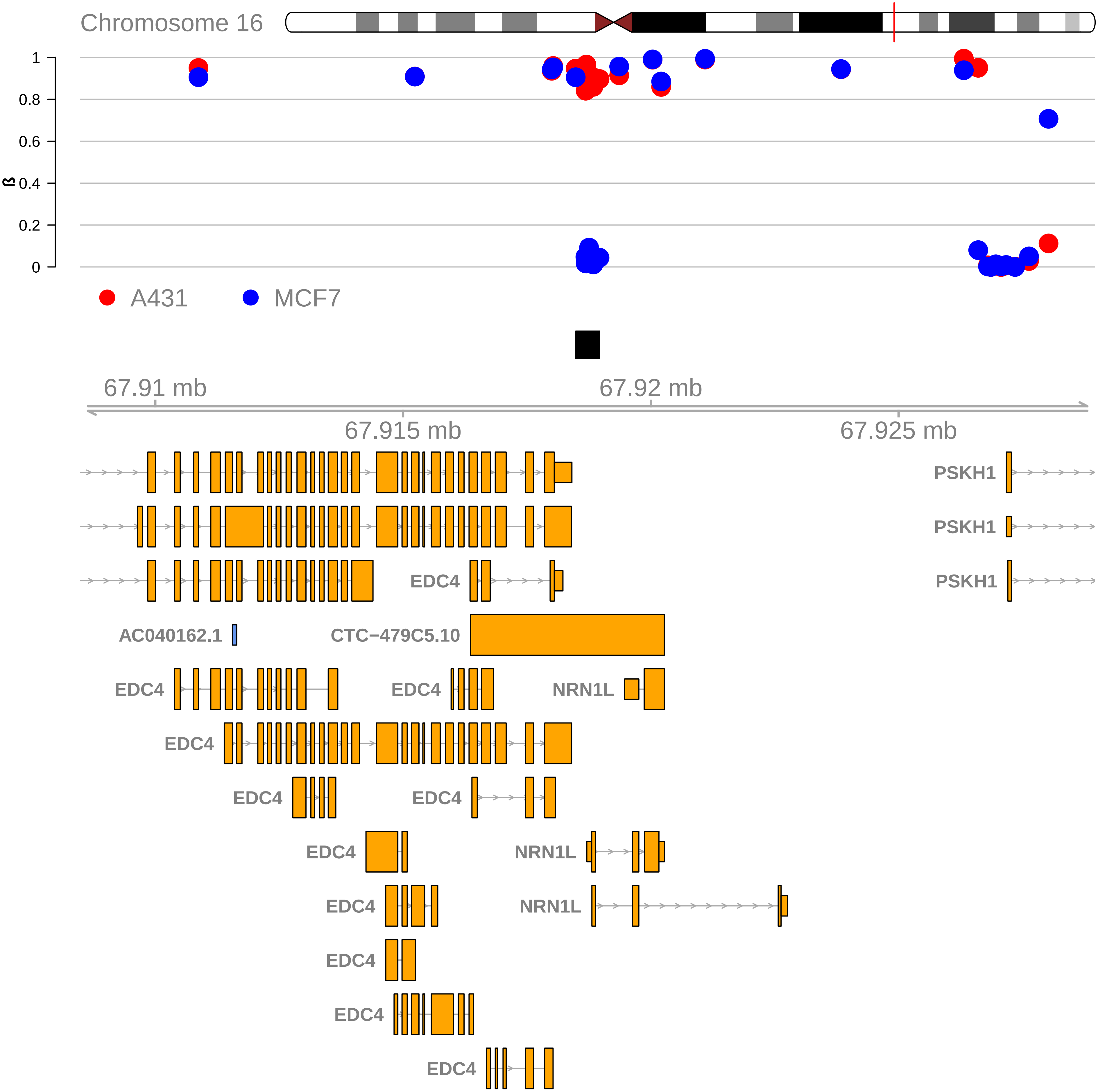

Chromosome 16
1
0.8
0.6
β 0.4
0.2
0
A431
MCF7
67.91 mb
67.92 mb
67.915 mb
67.925 mb
PSKH1
PSKH1
EDC4
PSKH1
AC040162.1
CTC−479C5.10
EDC4
EDC4
NRN1L
EDC4
EDC4
EDC4
EDC4
NRN1L
EDC4
NRN1L
EDC4
EDC4
EDC4
